# Unusually Thickened Ulnar Nerve and Lagophthalmos in Leprosy

**DOI:** 10.4269/ajtmh.2010.09-0628

**Published:** 2010-05

**Authors:** Ravindra Kumar Garg

**Affiliations:** Department of Neurology, Chhatrapati Shahuji Maharaj Medical University, Uttar Pradesh, Lucknow, India

## Case summary

**Figure 1.**
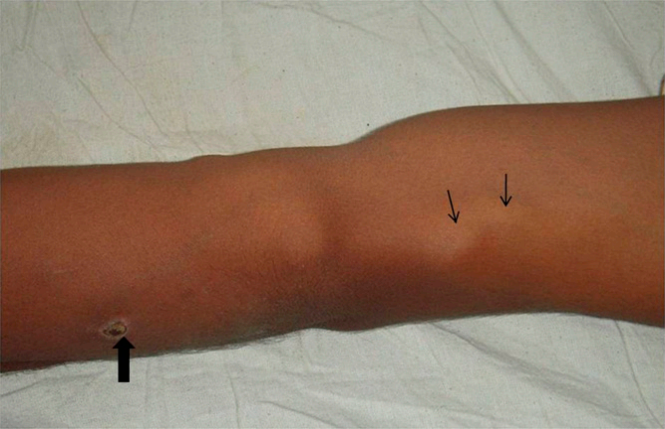
Unusually thickened ulnar nerve (fine arrows) in a patient with borderline leprosy. There is a hypopigmented skin lesion on the dorsal aspect of the elbow (a thick arrow). This figure appears in color at www.ajtmh.org.

**Figure 2.**
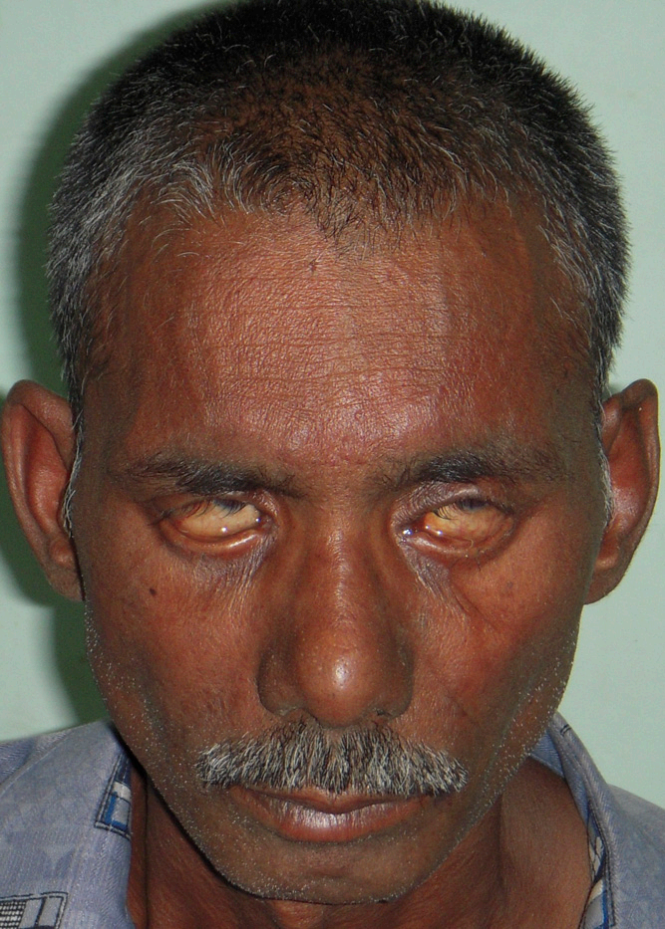
Lagophthalmos in a patient with lepromatous leprosy. Patient is attempting to close his eyelids but he is unable to close them. This figure appears in color at www.ajtmh.org.

Leprosy is a common cause of neuropathy. Thickened peripheral nerves are one of the cardinal features. Ulnar and common peroneal nerves are the most frequent thickened nerves.^1^ Figure 1 shows a visibly thickened ulnar nerve in a 22-year-old man presenting with mononeuritis multiplex along with hypopigmented and hypoesthetic skin lesions. Borderline leprosy has a high propensity to involve nerve trunks resulting in mononeuritis multiplex. Figure 2 shows lagophthalmos in a 45-year-old patient of lepromatous leprosy. Lagophthalmos is the inability to close the eye because of paralysis of the upper eyelid. Involvement of zygomatic and temporal branches of facial nerve result in lagophthalmos.^2^
